# A Bayesian spatio-temporal model for forecasting the prevalence of antibodies to *Ehrlichia* species in domestic dogs within the contiguous United States

**DOI:** 10.1186/s13071-017-2068-x

**Published:** 2017-03-09

**Authors:** Yan Liu, Robert B. Lund, Shila K. Nordone, Michael J. Yabsley, Christopher S. McMahan

**Affiliations:** 1 Department of Mathematical Sciences, Clemson University, Clemson, SC USA; 2 Department of Molecular and Biomedical Sciences, Comparative Medicine Institute, North Carolina State University, Raleigh, NC USA; 3 Department of Population Health, Southeastern Cooperative Wildlife Disease Study, College of Veterinary Medicine and the Warnell School of Forestry and Natural Resources, The University of Georgia, Athens, GA USA

**Keywords:** Autoregression, CAR Model, Ehrlichiosis, Head-banging, Kriging, Prevalence, Spatio-temporal modeling

## Abstract

**Background:**

Dogs in the United States are hosts to a diverse range of vector-borne pathogens, several of which are important zoonoses. This paper describes factors deemed to be significantly related to the prevalence of antibodies to *Ehrlichia* spp. in domestic dogs, including climatic conditions, geographical factors, and societal factors. These factors are used in concert with a spatio-temporal model to construct an annual seroprevalence forecast. The proposed method of forecasting and an assessment of its fidelity are described.

**Methods:**

Approximately twelve million serological test results for canine exposure to *Ehrlichia* spp. were used in the development of a Bayesian approach to forecast canine infection. Data used were collected on the county level across the contiguous United States from routine veterinary diagnostic tests between 2011–2015. Maps depicting the spatial baseline *Ehrlichia* spp. prevalence were constructed using Kriging and head-banging smoothing methods. Data were statistically analyzed to identify factors related to antibody prevalence *via* a Bayesian spatio-temporal conditional autoregressive (CAR) model. Finally, a forecast of future *Ehrlichia* seroprevalence was constructed based on the proposed model using county-level data on five predictive factors identified at a workshop hosted by the Companion Animal Parasite Council and published in 2014: annual temperature, percentage forest coverage, percentage surface water coverage, population density and median household income. Data were statistically analyzed to identify factors related to disease prevalence *via* a Bayesian spatio-temporal model. The fitted model and factor extrapolations were then used to forecast the regional seroprevalence for 2016.

**Results:**

The correlation between the observed and model-estimated county-by-county *Ehrlichia* seroprevalence for the five-year period 2011–2015 is 0.842, demonstrating reasonable model accuracy. The weighted correlation (acknowledging unequal sample sizes) between 2015 observed and forecasted county-by-county *Ehrlichia* seroprevalence is 0.970, demonstrating that *Ehrlichia* seroprevalence can be forecasted accurately.

**Conclusions:**

The forecast presented herein can be an a priori alert to veterinarians regarding areas expected to see expansion of *Ehrlichia* beyond the accepted endemic range, or in some regions a dynamic change from historical average prevalence. Moreover, this forecast could potentially serve as a surveillance tool for human health and prove useful for forecasting other vector-borne diseases.

## Background

*Ehrlichia* species are intracellular gram-negative bacteria that are maintained in a complex life-cycle involving vertebrate hosts as reservoirs and ticks as vectors [1–4]. In North America, multiple *Ehrlichia* spp. reportedly infect dogs, including *Ehrlichia canis*, *Ehrlichia chaffeensis*, *Ehrlichia ewingii*, *Ehrlichia* sp., Panola Mountain *Ehrlichia* (PME), or coinfection with multiple *Ehrlichia* species. Most of these bacteria (*E. chaffeensis*, *E. ewingii* and *Ehrlichia* sp. PME) are maintained in nature in white-tailed deer (*Odocoileus virginianus*) reservoirs and are transmitted by *Amblyomma americanum* (lone star ticks). *Ehrlichia canis* is primarily transmitted among domestic dogs by *Rhipicephalus sanguineus* (brown dog ticks). *Dermacentor variablis* (the American dog tick) is a potential vector of *E. chaffeensis* and *E. canis* [4–8]. In the absence of coinfection, *E. chaffeensis* produces relatively mild canine disease [4]; however, *E. chaffeensis* is most commonly cited as the causative agent in human monocytic ehrlichiosis [5]. While *E. canis* was historically believed to be the predominant *Ehrlichia* spp. to infect dogs, recent data on exposure of dogs to *Ehrlichia* spp. using species specific peptides has shed light on the spatial variation and prevalence of *Ehrlichia* exposure in dogs [7]. Qurollo et al. [7] found that in the Southern, Mid-Atlantic, Northeastern and Midwestern US dogs were predominantly exposed to *E. ewingii* and *E. chaffeensis*. In contrast, canine *E. canis* seroreactivity was low in these regions, and was the predominant, or only, *Ehrlichia* species responsible for *Ehrlichia* seroconversion in the western US.

Veterinary wellness exams commonly include annual screening for exposure to *Ehrlichia* spp.; *Anaplasma* spp., *Borrelia burdgorferi* (Lyme disease agent) and infection with *Dirofilaria immitis* (heartworm disease agent) using a rapid, in-house enzyme-linked immunosorbent assay (ELISA) platform (SNAP®3Dx®, SNAP® 4Dx® and SNAP®4Dx® Plus, IDEXX Laboratories, Inc.). This in-house assay, while highly specific and sensitive for exposure to *Ehrlichia* spp., uses recombinant peptides of major *E. canis* and *E. ewingii* outer membrane proteins [7, 8], thus precluding speciation of seroreactivity. As such, these tests are interpreted by veterinary clinicians to indicate tick exposure and a history of transmission of *Ehrlichia* spp. and possibly other tick-borne pathogens. Of four million dogs tested for exposure to *Ehrlichia* in 2015, over 100,000 dogs were seropositive for *Ehrlichia* spp. [9]. Clinical ehrlichiosis in dogs can manifest in one or more ways: acute, subclinical and chronic [10, 11]. The acute phase occurs within 1–3 weeks after tick transmission of *Ehrlichia* and includes enlarged lymph nodes, weakness, lethargy, depression, anorexia, labored breathing, and limb edema. Some dogs do not develop clinical signs of acute ehrlichiosis. After the acute phase dogs enter a subclinical phase in which infection may persist for months or years without clinical signs. Finally, during the chronic phase, dogs may experience abnormal bleeding such as epistaxis, become anemic, or have cyclic thrombocytopenia. They may also experience severe weight loss, fever, difficulty breathing due to lung inflammation, shifting leg lameness due to joint inflammation and pain, or kidney failure and paralysis [12].

Canine ehrlichiosis has been reported throughout the contiguous United States; however, the geographical range of different *Ehrlichia* spp. varies considerably, influenced by the range and density of their primary vectors. For example, the highest concentration of *E. canis* cases have been reported in southwestern and Gulf Coast regions of the United States, whereas the highest incidence of *E. chaffeensis* and *E. ewingii* cases occur in the midwestern and southeastern United States, places where *A. americanum* occurs in high densities. The distribution and number of ehrlichiosis cases have increased six-fold over the last five years [7, 8]; cases have been found in states as far north as Maine and as far west as Arizona, California and Nevada [9]. The dynamic change in prevalence in non-endemic regions has led to speculation on possible changing tick populations, which may be influenced by factors such as climate change, encroaching urbanization, and increasing urban/suburban populations of wildlife reservoirs.

In developing our approach, a Bayesian spatio-temporal conditional autoregressive (CAR) model is utilized to assess the putative factors and forecast future trends in *Ehrlichia* spp. prevalence. In this venue, Bayesian modeling offers a number of advantages over classical approaches [13–15]. First, the probabilistic likelihood-based methods here are highly flexible, and are able to adapt to data availability problems. The methods are also capable of assessing predictive significance of various covariate factors. Secondly, the use of data augmentation Markov-chain Monte Carlo (MCMC) methods to sample from a posterior distribution provides the opportunity to treat missing data, such as absence of serological data from certain counties, as latent (missing) variables, even in large populations [16, 17]. Finally, these methods are directly amendable to forecasting future trends in seroprevalence, conditional on the past history of data. The Bayesian methods capably quantify uncertainty both in terms of the potential stochasticity of the disease process and the model parameter estimates.

In what follows, eight factors previously purported to influence canine *Ehrlichia* seroprevalence will be examined: annual precipitation, annual relative humidity, annual temperature, elevation, percentage forest coverage, percentage surface water coverage, population density and median household income [18]. After a predictive model is developed from these factors, annual *Ehrlichia* seroprevalence forecasts are constructed and a comparison between the 2015 actual *versus* predicted *Ehrlichia* prevalence was conducted. Intended uses of annual *Ehrlichia* seroprevalence forecasts include: (i) to encourage the use of tick preventive to reduce exposure using an evidence-based tool, (ii) to promote the use of annual use of diagnostic testing in areas where the disease is emerging, and (iii) to potentially extend the use of canine data as a surveillance tool for physicians to assess potential threats to human health from *Ehrlichia* species.

## Methods

### The data and baseline map construction

The data included in this study were canine serological test results for antibodies to *Ehrlichia* spp. in the contiguous United States from 2011–2015, and various climate, geographical, and socio-economic factors purported to influence *Ehrlichia* seroprevalence. The dataset, obtained from IDEXX Laboratories, Inc. [19], contained 11,967,465 tests, 305,409 of which were positive (2.55%) for *Ehrlichia* spp. antibodies, and the county of the testing clinic. No information was available on demographic details of the individuals tested, such as age, sex or breed of dog, nor the travel or testing history of the dog, or the reason why the tests were conducted.

The test data were aggregated into the number of positive and negative tests for each year in each contiguous United States county or parish. The explanatory factors chosen for inclusion are those purported to be associated with canine *Ehrlichia* seroprevalence and for which data are readily available on a wide geographical scale in the United States [18]. Table 1 lists eight considered factors, the time period of recording, and the geographical scale of collection. These eight factors can be grouped into climatic variables (annual temperature, precipitation and relative humidity), geographical variables (county elevation, forestation coverage and surface water coverage), and socio-economic variables (population density and median household income), and are discussed in more detail in [20].

**Table 1:** Factors purported to influence *Ehrlichia* seroprevalence in domestic dogs. For further discussion, including the source of each factor, see [18, 20]

	Factor	Data period	Scale	Notation	Numerical scale of data
Climate factors	Annual temperature	1895–2015	Climate Division	*X*_*s,1*_ *(t)*	Continuous
	Annual precipitation	1895–2015	Climate Division	*X*_*s,2*_ *(t)*	
	Annual relative humidity	2006–2015	Climate Division	*X*_*s,3*_ *(t)*	
Geographical factors	Elevation	2012	County	*X*_*s,4*_ *(t)*	Continuous
	Percentage forest coverage	2012	County	*X*_*s,5*_ *(t)*	
	Percentage surface water coverage	2010	County	*X*_*s,6*_ *(t)*	
Societal factors	Population density	2011–2014	County	*X*_*s,7*_ *(t)*	Continuous
	Median household income	1997–2014	County	*X*_*s,8*_ *(t)*	

**Fig. 1:**
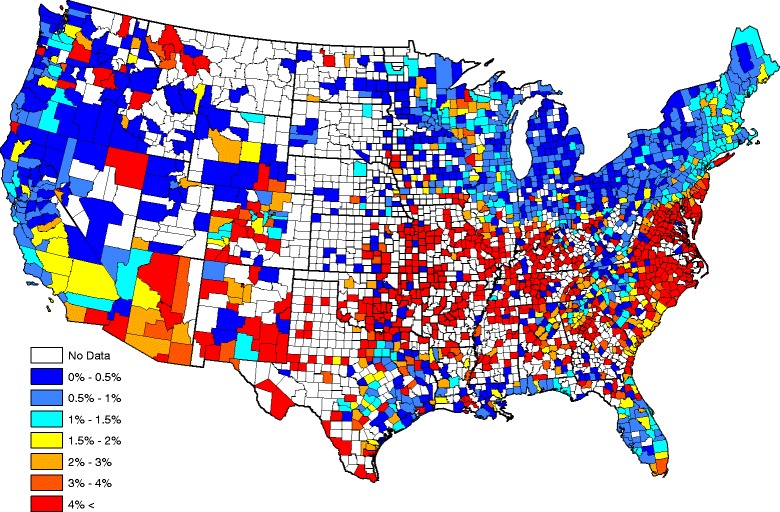
County level raw prevalences for *Ehrlichia* antibodies reported in domestic dogs, aggregated over 2011–2015

The county-by-county raw seroprevalence aggregated over the five-year data record are shown in Fig. 1. The raw seroprevalence in Fig. 1 exhibits apparent positive spatial dependence: counties near each other tend to report similar prevalences. Also, prevalence at a fixed county is often similar in adjacent years (this structure cannot be discerned from Fig. 1, but is evident from sample correlations and other diagnostics, data not shown); hence, positive temporal correlation exists in our data. In the next section, a statistical model that accounts for spatial and temporal correlation is developed to analyze these data.

**Fig. 2:**
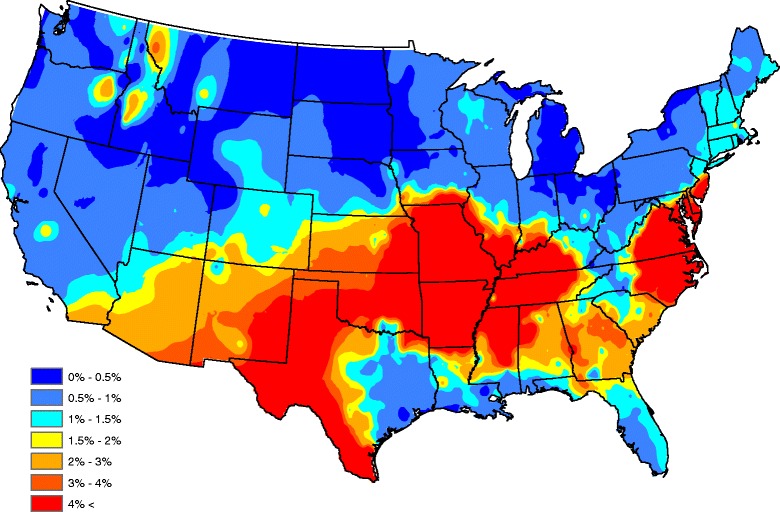
Head-banged baseline map showing *Ehrlichia* seroprevalences in domestic dogs for an average year

Figure 2 displays a map of the raw seroprevalences after a head-banging smoothing procedure was applied to produce a “baseline” (average year) map. Twenty triples and weights that are proportional to the number of observations taken in each county over the five-year period were used in the smoothing. Down weighting counties with a small number of tests helps account for sample size effects, preventing the map from signaling a high/low prevalence that is more likely attributed to a small sample size (e.g. one positive out of three tests has the same prevalence as one hundred positives in three hundred tests, though the latter has more certainty with respect to risk). Note, in order to provide a spatially complete map in Fig. 2, Kriging was implemented in ArcGIS using the default parameters. In general, Kriging is a standard spatial interpolation method for which the interpolated values are modeled by a Gaussian process. The predictions obtained using this technique are based on the assumptions that spatial variability in the data is related to the distance between observations, and that values in unsampled areas can be predicted as a weighted average of observations at nearby locations.

The map in Fig. 2 shows a band of high prevalence stretching from western Texas and eastern New Mexico, through northern Texas, eastern Oklahoma, Arkansas, Missouri, northern Mississippi, and western Tennessee. Another zone of high prevalence is seen in eastern North Carolina and Virginia; this zone ceases over the southern Appalachian Mountains. Other isolated high-prevalence regions occur in central Georgia and the northern Rockies. The western United States generally has lower prevalence compared with the southeastern United States, but isolated foci of seropositive dogs exist in many areas of the country. Several factors, including habitat, hosts, microclimate, etc., may be associated with the presence and location of theses isolated foci [21] but nonetheless, Fig. 2 should serve as an accurate depiction of the baseline ehrlichiosis risk for dogs.

## Results

### Model

The model and methods used to statistically analyze the *Ehrlichia* test results are now described. The goal is to assess which of the proposed factors significantly influence *Ehrlichia* presence and whether they increase or decrease prevalence.

Let *Y*_*s*_(*t*) and *n*_*s*_(*t*) denote the number of positive and total tests conducted in year *t* and county *s*, respectively, for counties *s* = 1, …, *S* and years *t* = 1, …, *T*. Bayesian hierarchical models have successfully described many spatial-temporal dependent data sets. Here, spatial and temporal dependence is modeled in a hierarchy by introducing random effects with certain structures. For a modern review of spatio-temporal models, see [22, 23]. Typically, a Poisson marginal distribution is preferred to model spatial or spatial-temporal dependent count data [23–26]. Motivated by [24], the following Bayesian hierarchical Poisson regression model is adopted:

1 $$ {Y}_s(t)\Big|{n}_s(t),{p}_s(t)\kern0.62em \sim \kern0.62em \mathrm{Poisson}\left\{{n}_s(t){p}_s(t)\right\}, $$

2 $$ \log \left\{{p}_s(t)\right\}\kern0.62em =\kern0.62em {\beta}_0+{\displaystyle \sum_{k=1}^8}{\beta}_k{X}_{s, k}(t)+{\xi}_s(t), $$

where log(⋅) denotes natural logarithm, ∼ means has the distributional type, **X**_*s*_(*t*) = (*X*_*s*,1_(*t*), …, *X*_*s*,8_(*t*))' contains the factor information at the time *t* (' denotes matrix transpose), *p*_*s*_(*t*) denotes the prevalence of *Ehrlichia* within county *s* at time *t*, and the symbol ∣ indicates given quantity(ies). For example, the first equation above indicates that *Y*_*s*_(*t*) has a Poisson distribution with mean *n*_*s*_(*t*)*p*_*s*_(*t*) when one knows that *n*_*s*_(*t*) tests were taken in county *s* during year *t* and the prevalence at this county and time is *p*_*s*_(*t*). The quantities **β** = (*β*_0_, …, *β*_*p*_)' are factor regression coefficients.

Equation (2) induces spatial and temporal correlation in our model through the random effects *ξ*_*s*_(*t*), for *s* = 1, …, *S* and *t* = 1, …, *T*. The disease counts are assumed to be conditionally independent across counties given the factors and random effects - without additional information, this is a reasonable (and standard) assumption.

There are several common ways to induce spatial and temporal dependence with the random effects {*ξ*_*s*_(*t*)}. The autoregressive structures

3 $$ {\xi}_1\kern0.62em =\kern0.62em {\boldsymbol{\phi}}_1; $$

4 $$ {\xi}_t\Big|{\xi}_{t-1},\varphi \kern0.62em =\kern0.62em \varphi {\xi}_{t-1}+{\boldsymbol{\phi}}_t,\mathrm{f}\mathrm{o}\mathrm{r}\  t=2,\dots, T; $$

5 $$ {\boldsymbol{\phi}}_t\kern0.62em \sim \kern0.62em \mathrm{CAR}\left({\tau}^2;\rho \right),\mathrm{f}\mathrm{o}\mathrm{r}\  t=1,\dots, T, $$

are natural choices. Here, **ξ**_*t*_ = (*ξ*_1_(*t*), …, *ξ*_*S*_(*t*))' and ***ϕ***_*t*_ = (*ϕ*_1_(*t*), …, *ϕ*_*S*_(*t*))' are autoregressive and CAR effects at time *t*. Elaborating, (5) uses a conditional autoregressive distribution [27], which is a popular approach to model spatial dependence (see [28]). In this setup, the spatial random effects ***ϕ***_*t*_ at time *t* are independent and identically distributed in time, and for each fixed time, follow a CAR distribution across the counties, which is a spatially correlated random field.

More specifically, let ***ϕ*** = (*ϕ*_1_, …, *ϕ*_*S*_)' denote a random vector drawn from our CAR distribution at any fixed year. Such an object contains an effect for each county. Typically, the distribution of ***ϕ*** is specified through a set of *S* univariate conditional distributions; spatial dependence is induced in a neighboring system involving geographically adjacent counties. Our CAR version, taken from [29], uses

6 $$ \begin{array}{cc}\kern1em {\phi}_s\mid {\boldsymbol{\phi}}_{- s},{\tau}^2,\rho, \mathbf{W}\sim \mathrm{N}\left(\rho \frac{{\displaystyle {\sum}_{s^{\prime}\ne s}}{w}_{s,{s}^{\prime }}{\phi}_{s^{\prime }}}{{\displaystyle {\sum}_{s^{\prime}\ne s}}{w}_{s,{s}^{\prime }}},\frac{\tau^2}{{\displaystyle {\sum}_{s^{\prime}\ne s}}{w}_{s,{s}^{\prime }}}\right),\mathrm{for}\  s=1,\dots, S,\kern1em & \kern2em \end{array} $$

where ***ϕ***_*-s*_ = (*ϕ*_1_, …, *ϕ*_*s* − 1_, *ϕ*_*s* + 1_, …, *ϕ*_*S*_)'   contain effects for all counties except the *s*th one and *N*(*μ*, *σ* ^2^) denotes a normally distributed quantity with mean *μ* and variance *σ* ^2^. Here, **W** = {*w*_*s*,*s'*_} is an *S* × *S* dimensional matrix whose entries are either zero or unity. Specifically, *w*_*s*,*s'*_ is taken as unity (i.e. *w*_*s*,*s'*_ = 1) if and only if the *s*th and *s'*  th counties border each other (at some place); *w*_*s*,*s'*_ = 0 otherwise. The parameter *τ* ^2^ is a scaling variance parameter and *ρ* ∈ [0, 1] is the autocorrelation between bordering counties. When *ρ* = 0, the *ϕ*_*s*_ are independent over different counties *s*; antipodally, *ρ* close to unity indicates strong spatial dependence in bordering counties.

In (4), a temporal autoregressive model of order one (AR(1)) is used to describe temporal dependence. The AR(1) model, a time series staple [30], describes the prevalence at each fixed county across different years. The parameter *φ* is the temporal correlation between consecutive years and lies within (-1, 1). This ensures a causal and stationarity solution to the time series model (see [30]), which is needed in estimation.

From (6), the conditional expectation of *ϕ*_*s*_ given its neighbor’s random effects (and other parameters) is the scaled (by *ρ*) weighted average of the neighboring random effects. The conditional variance of *ϕ*_*s*_ given its neighbor’s random effects is inversely proportional to the number of its neighbors. Hence, counties that border many other counties have a smaller variance. This is intuitive as data from bordering counties are useful with respect to predicting the prevalence of a given county, and more data equates more precise predictions. From (6), the joint distribution of ***ϕ*** can be shown to be multivariate normal:

7 $$ \boldsymbol{\phi} \kern0.62em \sim \kern0.62em \mathrm{N}\left(0,\boldsymbol{\Gamma} \right); $$

8 $$ \boldsymbol{\Gamma} \kern0.62em =\kern0.62em {\tau}^2{\left(\mathbf{D}-\rho \mathbf{W}\right)}^{-1}, $$

where **W** is the aforementioned neighborhood matrix and **D** = {*d*_*s*,*s'*_} is an *S* × *S* diagonal matrix whose *s*th diagonal element is the number of counties that county *s* borders: ${d}_{s, s}={\displaystyle \sum_{s^{\prime}\ne s}}{w}_{s,{s}^{\prime }}$ (*w*_*s*,*s*_ = 0 here).

The parameters to be estimated include **β**, *φ*, *ρ*, and *τ* ^2^. In order to complete the Bayesian model, prior distributions for these parameters were specified as:

9 $$ {\beta}_k\kern0.62em \sim \kern0.62em \mathrm{N}\left(0,1000\right),\mathrm{f}\mathrm{o}\mathrm{r}\kern0.5em  k=0,\dots, 8; $$

10 $$ \varphi \kern0.62em \sim \kern0.62em \mathrm{Uniform}\left(-1,1\right); $$

11 $$ \rho \kern0.62em \sim \kern0.62em \mathrm{Uniform}\left(0,1\right); $$

12 $$ {\tau}^{-2}\kern0.62em \sim \kern0.62em \mathrm{Gamma}\left(0.5,0.05\right). $$

Note, the prior distributions for *φ* and *ρ* are uninformative (all admissible possibilities are equally likely); those for the regression coefficients *β*_0_, …, *β*_8_ are diffuse (so that inferences for these parameters are based mainly on the data); and the prior for *τ* ^- 2^ is chosen as a conjugate-prior (the posterior and prior distributions are from the same distributional family) for ease of computation. The random effects and model parameters are estimated based on posterior samples from a Markov chain Monte Carlo (MCMC) simulation. The MCMC simulation for our model uses a combination of Gibbs and Metropolis-Hastings steps [13, 16, 17]. In the algorithm, prevalence estimates for counties not reporting any data are infilled for ease of computation. To run our MCMC simulation and assess factor significance, a program was developed and implemented in R and C++.

### Factor assessment

Eight explanatory factors are available for inclusion in the spatio-temporal Poisson regression model in (1). To assess which factors significantly influence *Ehrlichia* seroprevalence, a “full" model with all eight factors was first fitted and credible intervals for all regression parameters were calculated from the MCMC posterior samples. Table 2 shows estimates of these eight regression coefficients (posterior median) and their 98.75% highest posterior density (HPD) intervals. These HPD intervals were adjusted to account for multiple comparisons using the standard Bonferroni correction with a family wise error rate of 10%. For HPD interval details, see [13].

**Table 2:** Parameter estimates from the full model

Factor	Estimate	98.75% HPD Interval
Annual temperature	0.022	[0.003, 0.042]
Annual precipitation	-0.008	[-0.060, 0.049]
Annual relative humidity	-0.004	[-0.010, 0.004]
Elevation	0.025	[-0.001, 0.056]
Percentage forest coverage	3.295	[2.171, 4.499]
Percentage surface water coverage	0.519	[0.173, 0.804]
Population density	-3.578 × 10^-5^	[-5.692 × 10^-5^, -1.301 × 10^-5^]
Median household income	-0.003	[-0.007, -0.001]

The HPD intervals in Table 2 show that not all factors are significant; for example, credible intervals for annual precipitation, annual relative humidity, and elevation contain zero. As a parsimonious model with only significant factors is desired, eight models were fitted, each containing a combination of the three questionable factors of annual precipitation, annual relative humidity, and elevation, along with the other five factors. In all additional model fits, these three factors were deemed insignificant. To further investigate these three insignificant factors, three additional models were fit, each using only the dismissed factor as the only covariate. From these analyses, it was judged that each of these factors were indeed insignificant. In particular, 95% HPD intervals associated with the regression coefficients for annual precipitation, annual relative humidity, and elevation were [-0.057, 0.035], [-0.007, 0.002] and [-0.013, 0.038], respectively. Therefore, our parsimonious model includes the five factors of annual temperature, percentage forest coverage, percentage surface water coverage, population density, and median household income. Parameter estimates (posterior median) and 95% HPD intervals for the regression parameters in this model are presented in Table 3. The estimates (posterior median) of the other model parameters are *φ* = 0.893, *ρ* = 0.999, and *τ* ^2^ = 0.574.

**Table 3:** Parameter estimates from the selected model

Factor	Estimate	95% HPD Interval
Annual temperature	0.021	[0.007, 0.030]
Percentage forest coverage	3.276	[2.407, 4.223]
Percentage surface water coverage	0.458	[0.242, 0.718]
Population density	-3.578 × 10^-5^	[-5.123 × 10^-5^, -1.789 × 10^-5^]
Median household income	-0.004	[-0.006, -0.001]

**Fig. 3:**
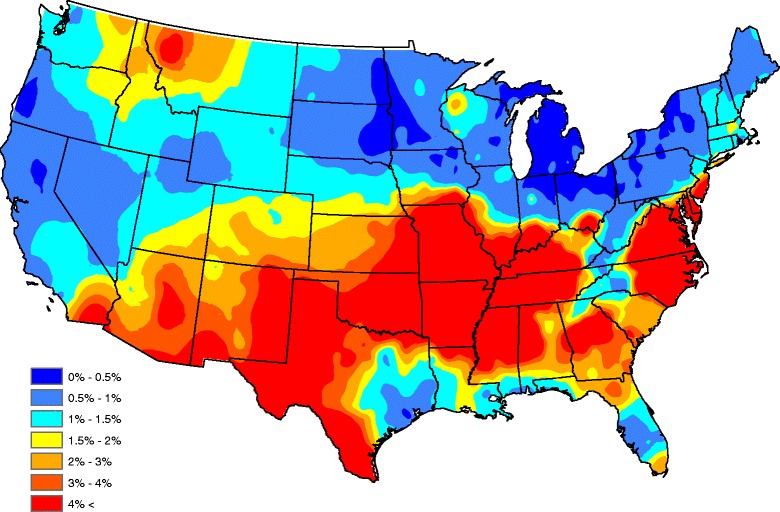
Model-based *Ehrlichia* seroprevalences

Our parsimonious five factor model in Table 3 shows that *Ehrlichia* seroprevalence increases with increasing annual temperature, forest coverage, and surface water coverage while seroprevalence decreases with increasing population density and median household income. Figure 3 graphically portrays our fitted model by plotting the average model-predicted prevalence (over all years) after smoothing (Kriging with default parameters were used in the software ArcGIS). The picture compares well to the head-banging smoothed baseline in Fig. 2. In fact, the correlation between the Figs. 2 and 3 graphics is 0.842 (this correlation is taken over counties reporting at least one test during the five year study period). Our correlation between the two observation sets {*A*_*s*_}_*s* = 1_^*S*^ and {*B*_*s*_}_*s* = 1_^*S*^ is

13 $$ \mathrm{Corr}\left(\left\{{A}_s\right\},\left\{{B}_s\right\}\right)=\frac{{\displaystyle {\sum}_{s=1}^S}{n}_s\left({A}_s-\overline{A}\right)\left({B}_s-\overline{B}\right)}{\sqrt{{\displaystyle {\sum}_{s=1}^S}{n}_s{\left({A}_s-\overline{A}\right)}^2{\displaystyle {\sum}_{s=1}^S}{n}_s{\left({B}_s-\overline{B}\right)}^2}}, $$

where

$$ \overline{A}=\frac{{\displaystyle {\sum}_{s=1}^S}{n}_s{A}_s}{{\displaystyle {\sum}_{s=1}^S}{n}_s},\overline{B}=\frac{{\displaystyle {\sum}_{s=1}^S}{n}_s{B}_s}{{\displaystyle {\sum}_{s=1}^S}{n}_s} $$

are the sample-size weighted averages of {*A*_*s*_}_*s* = 1_^*S*^ and {*B*_*s*_}_*s* = 1_^*S*^, and *n*_*s*_ is the number of tests conducted in county *s*. Note, the expression in (13) denotes the usual weighted sample correlation and directly accounts for sample size differences (i.e. different values of *n*_*s*_). Because the correlation here is between smoothed and model-estimated prevalence (these are not sample size dependent quantities), the weights were taken as *n*_*s*_≡1, which causes (13) to revert to the usual sample correlation. The 0.842 correlation achieved indicates that the regression model has explained most of the data’s structure.

The fitted model has a number of uses. In the next section, the model is used to construct an annual *Ehrlichia* seroprevalence forecast. The model could also be used to extrapolate how climate change or other changes in factors would influence ehrlichiosis risk.

### Forecasting

This section builds an annual *Ehrlichia* seroprevalence forecast from the model in the last section. Such a forecast can be used to issue alerts or make veterinarians aware of areas expected to be problematic in advance. Remediation tactics could be based on the forecasts.

To forecast next year’s *Ehrlichia* seroprevalence, all five significant explanatory factors and the spatial-temporal effects will need to be forecasted. Two of the five factors are relatively stable: county forestation and surface water coverage do not change appreciably in time. Hence, the most recent observations of these factors are used as next year’s forecasted values.

To forecast annual temperature, historical temperature records were collected from 1895 to 2015 for each county and modeled as an autoregressive model of order one (AR(1)). The AR(1) model for an annual temperature series {*F*_*t*_} (previously denoted by {*X*_*s*,1_(*t*)} for county *s*) obeys the difference equation

$$ {F}_t=\delta +\gamma {F}_{t-1}+{\omega}_t, $$

where {*ω*_*t*_} is zero mean white noise; for further details on time series and forecasting, see [30]. The AR(1) model can be fit to the observations in {*F*_*t*_} using practically any statistical software package. Let $\widehat{\delta}$ and $\widehat{\gamma}$ denote estimates of *δ* and *γ*, respectively. A prediction of the annual temperature at year *t* + 1 from temperatures from year 1 to year *t* is

$$ {\widehat{F}}_{t+1}=\widehat{\delta}+\widehat{\gamma}{F}_t. $$

**Fig. 4:**
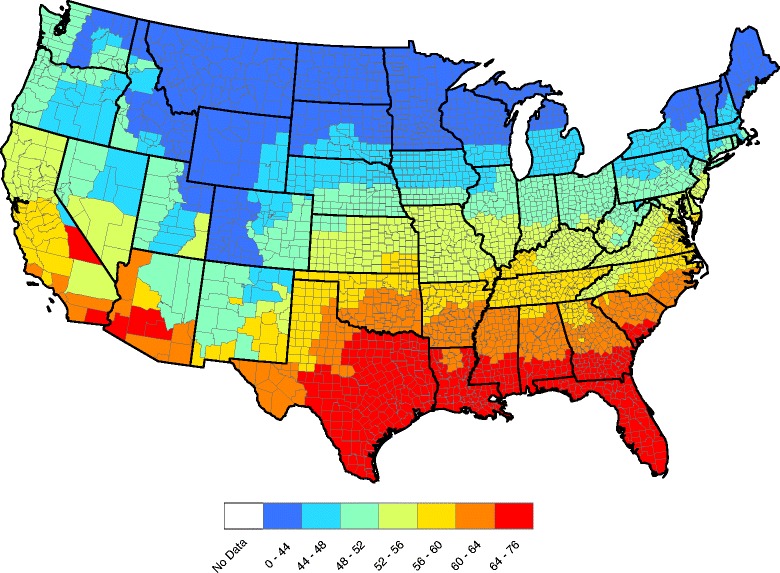
County-by-county forecasted 2015 annual average temperatures (°F)

**Fig. 5:**
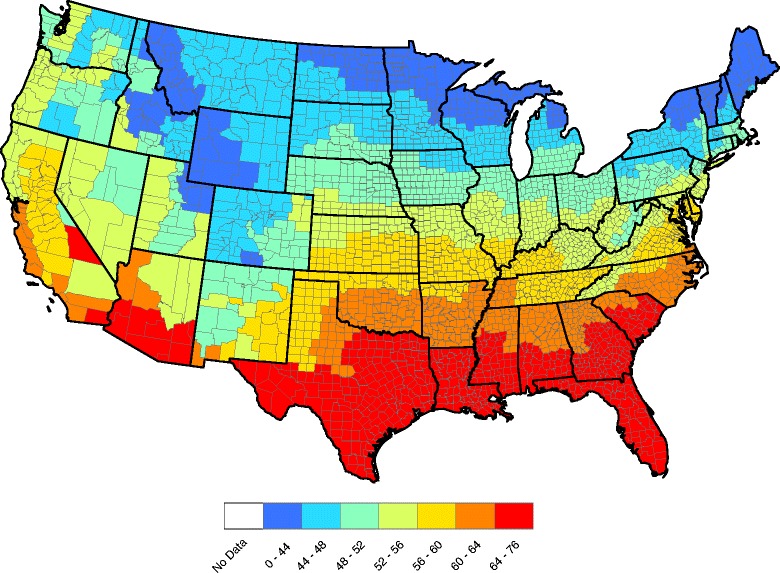
County-by-county observed 2015 annual average temperatures (°F)

In our forecast, ${\widehat{F}}_{t+1}$ is used at next year’s forecasted factor value. Figures 4 and 5 show forecasted and observed annual temperatures for 2015. The correlation between these two figures in (13) is *r* = 0.996, which suggests a high degree of fidelity in our predictive model.

A simple linear regression model was used to forecast next year’s median household income {*I*_*t*_} in each county (this factor was previously denoted by {*X*_*s*,8_(*t*)}. Historical median household incomes from 1997–2014 were used to fit the regression model in each county:

$$ {I}_t=\alpha +\kappa t+{\eta}_t, $$

where {*η*_*t*_} is zero mean random noise. Least squares estimators of *α* and *κ*, denoted by $\widehat{\alpha}$ and $\widehat{\kappa}$, respectively, were computed from the data at each county. The forecasted value for year *t* + 1 (year 2015) is simply

$$ {\widehat{I}}_{t+1}=\widehat{\alpha}+\widehat{\kappa}\left( t+1\right). $$

Forecasting the county population density for next year requires the county areas and their recent population counts. The US Census provides reliable county population counts for 2010, but not every year since then. Estimated state populations were obtained for each state between 1969–2014. A simple linear regression was fitted to this data for each state and 2015 state populations were forecasted. This forecasted state population was then partitioned to the counties within the state at a proportion that agrees with 2010 Census.

To forecast the spatial and temporal random effects a year in advance, formula (4) is used. Since the ***ϕ***_*t*_ s are independent and identically distributed over various years, given values of *τ* ^2^ and *ρ* (available from the posterior samples), ***ϕ***_*t* + 1_ is generated randomly from the multivariate normal distribution N(0, *τ* ^2^(**D** − *ρ* **W**)^- 1^). Then **ξ**_*t* + 1_ is set to **ξ**_*t* + 1_ = *φ* **ξ**_*t*_ + ***ϕ***_*t* + 1_. This process is repeated for each pair of *ρ* and *τ* ^2^ available from the posterior sample, thus yielding a sample of the next year’s random effects. See [14] for additional detail.

To see how our forecast performs, the 2015 test and factor data were removed from the analysis, and the proposed model was refitted with data from 2011–2014 only. Forecasts are based on use of the five significant factors annual temperature, percent forest coverage, percent surface water coverage, population density, and median household income (also used is are generated random effects for 2015).

**Fig. 6:**
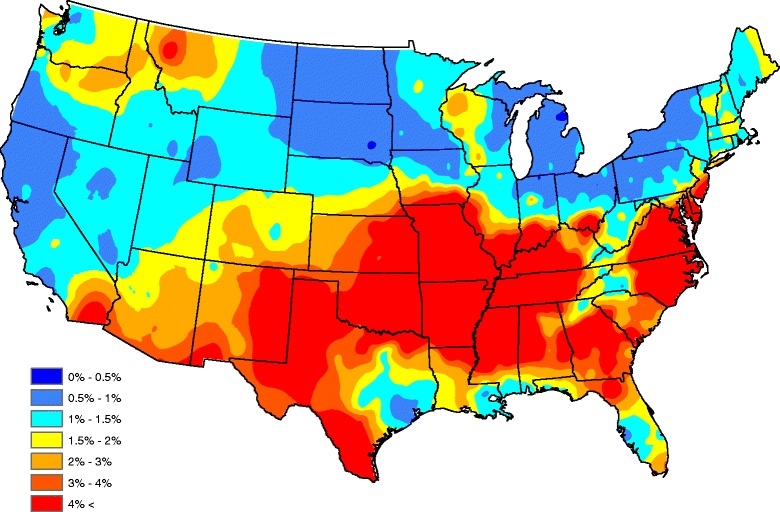
Observed *Ehrlichia* seroprevalence in domestic dogs for 2015

**Fig. 7:**
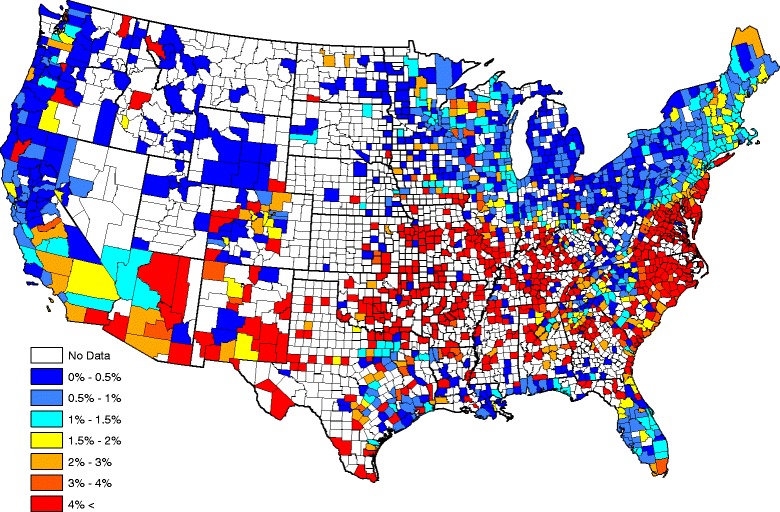
Forecasted *Ehrlichia* seroprevalence in domestic dogs for 2015

**Fig. 8:**
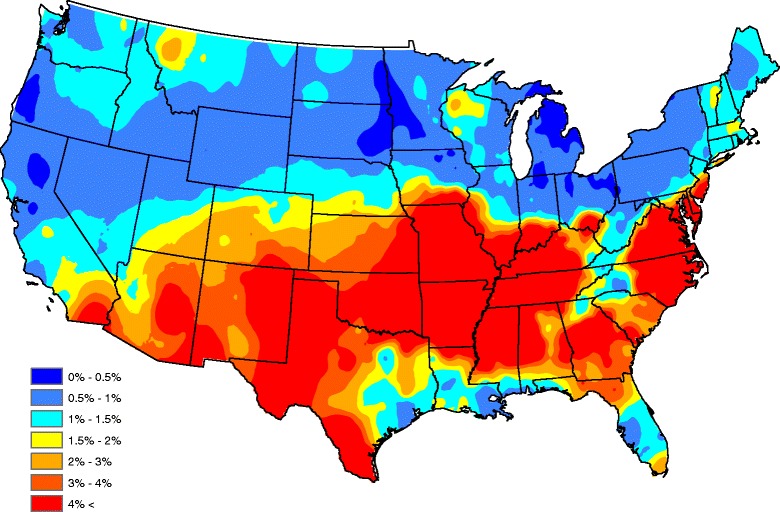
Forecasted *Ehrlichia* seroprevalence in domestic dogs for 2016

Figures 6 and 7 compare observed and forecasted *Ehrlichia* seroprevalences during 2015. The correlation between the two maps is 0.97 (this is a weighted correlation computed according to (13), where *n*_*s*_ denotes the number of tests performed within each county during 2015) and indicates significant skill in forecasting *Ehrlichia* seroprevalence in dogs one year in advance. One can get an idea where *Ehrlichia* seroprevalence is forecasted to be higher/lower than average by comparing Figs. 2 and 7. Figure 8 presents our *Ehrlichia* forecast for 2016 (this uses all data and factors from 2011–2015). When 2016 concludes, a future study will compare our current forecast with actual data.

## Discussion

In this paper, the first Bayesian approach to forecasting and inference for canine *Ehrlichia* seroprevalence, in the absence of detailed information on vector ecology, was made. While vector factors such as distribution and abundance are no doubt important, annual counts of all possible vector populations are currently economically and logistically infeasible to collect. Such data deficiencies will likely continue to hinder development of dynamic micro-scale vector-borne disease models, necessitating development of novel approaches to disease surveillance. As such, we have developed a model for forecasting spatial and temporal patterns of risk of exposure to *Ehrlichia* spp. based on canine seroprevalence data. Data used in this study indicate exposure to *Ehrlichia* spp. [19], but unfortunately lack the detailed molecular assessment that would allow species-level identification. Also, our data are obtained from a commercial diagnostic lab to which veterinarians had submitted samples, and as such, these dogs were acquiring veterinary care. This suggests that our data are a conservative estimate of the prevalence in domestic dogs because dogs at the highest risk of tick exposure would be dogs that receive no veterinary care, those from lower socioeconomic families, or are owned by clients who refuse these additional tests during well visits. Additionally, a lack of education of tick-borne pathogens may limit testing that veterinarians request; however, these tests are often run during routine heartworm testing so this latter issue is likely a minimal concern. Despite these limitations, these data are acquired on a monthly basis [9], providing a robust and timely source of information about the dynamic change of canine *Ehrlichia* spp. seroprevalence across the contiguous US, and holds promise for longitudinal studies to best understand the dynamic nature of vector-borne disease over time. From five years of historical diagnostic tests, our data show that a Bayesian model can capably quantify *Ehrlichia* seroprevalence, which ultimately will support qualitative decision-making and surveillance in disease management and response. When comparing actual to forecasted *Ehrlichia* seroprevalence in 2015, a weighted correlation of 0.97 was achieved, demonstrating significant predictive skill. In the future, we hope to gauge the effectiveness of interventions designed to reduce the occurrence of ehrlichiosis.

Of the eight factors evaluated as infection drivers, increasing annual temperature, percentage forest coverage, and percentage surface water coverage were deemed to increase prevalence, and increasing population density and median household income act to decrease prevalence. In general, these results support the presumption that increasing urbanization would decrease appropriate tick habitat. These factors also would potentially impact white-tailed deer, the primary vertebrate wildlife reservoir hosts of *E. chaffeensis* and *E. ewingii*; however, they are adaptable and do well across a range of urbanization other than highly urban. Domestic dogs, the natural host of *E. canis* and also long-term hosts for *E. ewingii* [31], would be present across all of the urbanization zones, but more rural or suburban households are likely to have outdoor dogs which would have a higher risk of tick exposure. Also, most of these factors are similar to those judged influential in the incidence of human monocytic ehrlichiosis and Rocky Mountain spotted fever [32, 33]. Interestingly, ambient humidity was not judged to be a significant factor in our model (in contrast to [33] for human monocytic ehrlichiosis, by [32] for Rocky Mountain spotted fever cases, and by [34] for *E. chaffeensis* exposure in white-tailed deer). The statistical methods utilized in the three aforementioned studies, along with their spatial scales, are definitively different than those of our study. Despite these differences, the contrasting results are more likely explained by the impact of forest coverage and resulting leaf litter layer on the ability of *Amblyomma americanum* ticks, the vector of *E. chaffeensis* and *E. ewingii* to undergo interstadial development in the environment [35]. Moreover, the logistic regression analysis for prevalence of antibody to *Ehrlichia* in deer [34], authors found that relative humidity was only significant for the eastern US and only during the summer months which corresponds with the spatial and temporal activity of *A. americanum*. On a local or regional scale, relative humidity may have been included in models, but humidity is likely associated with other factors already included in our model. Percentage forest coverage was judged as a significant factor of *Ehrlichia* seroprevalence in our model. We consider this to be important because the interstadial development of ticks vectors of *E. ewingii* and *E. chaffeensis* occurs within leaf litter refuges. Therefore, the rate of tick development is less likely to change in response to short term variability in ambient humidity, but rather by the presence or absence of refuge habitat conducive to tick survival [36]. Ultimately, aggregating factors into yearly summaries, as is necessary for our analysis, may hide the association between factors previously purported to influence tick populations (e.g. annual precipitation and humidity).

As previously discussed, canine ehrlichiosis is well-recognized as endemic in much of the southern half of the US, including the Southeast, Midwest and Mid-Atlantic states. The *Ehrlichia* seroprevalence forecast for 2016 in Fig. 8 suggests some changes in local prevalence. The forecast suggests an increase in prevalence throughout southern Indiana and Ohio, and an increased prevalence in central South Carolina, central Georgia and northern Florida. This coincides with recently reported increases in the distribution of *A. americanum*, an important vector of some *Ehrlichia* spp. in Indiana and Ohio [35, 36]. Although this tick species is generally widespread in South Carolina, Georgia and Florida, our 2016 forecasted increase may be due to climate-related increases in tick abundance. In the western US, the forecast predicts increased prevalence in western Texas, eastern Arizona, and eastern New Mexico, and encroachment into southern California. In these regions, *E. canis* is the predominant species associated with canine ehrlichiosis and the predicted increases may relate to the changing ecology of *R. sanguineus* in the region or some other unknown factor. In recent years, an increase in Rocky Mountain spotted fever in humans and dogs following exposure to *Rickettsia rickettsii* transmitted by *R. sanguineus* has been reported in Arizona [37–40]. These predicted changes motivate an increased dialog between pet owners and veterinarians to enhance timely diagnosis and year-round use of tick preventive.

Though the proposed technique could be used to construct long-term forecasts, caution should be taken. In particular, our approach makes use of forecasted values of the significant factors, with some factors being assumed to be static throughout time (e.g. forestation and surface water coverage). This assumption is reasonable in the short-term, but would obviously be problematic over a much larger time span, e.g. twenty to fifty years. Moreover, in general, when forecasting future trends one should be cautious of long-term forecasts, due to possible violations of assumed model forms not apparent in the available data, e.g. median household income is increasing/decreasing linearly throughout time. Thus, we promote the use of our approach to provide only short-term forecasts of spatial trends in *Ehrlichia* spp. seroprevalence.

Finally, an association between canine *Ehrlichia* seroprevalence and human ehrlichiosis is gaining appreciation by the Centers for Disease Control and Prevention. As discussed in a 2016 Morbidity and Mortality Weekly Report [41], dogs are frequently exposed to ticks due to their close contact with the environment and are susceptible to infections with many of the same tick-borne pathogens as humans, including *R. rickettsii*, *E. chaffeensis*, *E. ewingii* and *A. phagocytophilum*. Further, the CDC recognizes that “tick-infested dogs can transfer ticks directly to humans during interactions and serve as transport hosts, carrying ticks in and around dwellings where the ticks can then transfer to the human occupants”. The CDC recommends physicians question patients about contact with pets, especially dogs, and a history of tick attachment or recent tick removal from pets when assessing human exposure. Finally, clustering of tick-borne diseases is common, and infection with *Ehrlichia* species and several other vector-borne pathogens such as *Rickettsia* and *Bartonella* have been concurrently observed in humans and pet dogs [42–44]. Given that the majority of canine seroreactivity to *Ehrlichia* spp. is specific for *E. ewingii* and *E. chaffeensis* [7], communication between veterinarians and physicians is of critical importance when zoonotic diseases are suspected.

## Conclusions

We provide the first report of a Bayesian approach to forecasting and inference for canine *Ehrlichia* seroprevalence, in the absence of detailed information on vector ecology. The information provided promotes a better understanding of the expansion of *Ehrlichia* spp. beyond their accepted endemic range, or in some regions a dynamic change from historical average prevalence. The forecast can potentially inform public or veterinary health about *Ehrlichia* spp. in their area as well as information regarding the possible consequences of ecological changes on the range and prevalence of *Ehrlichia* spp. infections.
